# Common and unique associated factors for medically unexplained chronic widespread pain and chronic fatigue^☆^

**DOI:** 10.1016/j.jpsychores.2015.10.004

**Published:** 2015-12

**Authors:** J. McBeth, B. Tomenson, C.A. Chew-Graham, G.J. Macfarlane, J. Jackson, A. Littlewood, F.H. Creed

**Affiliations:** a Arthritis Research UK Epidemiology Unit, The University of Manchester, Manchester, UK; b Biostatistics Unit, Institute of Population Health, The University of Manchester, Manchester, UK; c Research Institute, Primary Care and Health Sciences, Keele University, Newcastle, Staffs, ST5 5BG, UK; d Musculoskeletal Research Collaboration (Epidemiology Group), School of Medicine, Medical Sciences and Nutrition, University of Aberdeen, UK; e Institute of Brain, Behaviour and Mental Health, University of Manchester, UK

**Keywords:** Chronic fatigue, Epidemiology, Fibromyalgia, Functional somatic syndromes, Medically unexplained symptoms, Population based

## Abstract

**Objective:**

Chronic widespread pain and chronic fatigue share common associated factors but these associations may be explained by the presence of concurrent depression and anxiety.

**Methods:**

We mailed questionnaires to a randomly selected sample of people in the UK to identify participants with chronic widespread pain (ACR 1990 definition) and those with chronic fatigue. The questionnaire assessed sociodemographic factors, health status, healthcare use, childhood factors, adult attachment, and psychological stress including anxiety and depression. To identify persons with *unexplained* chronic widespread pain or *unexplained* chronic fatigue; we examined participant's medical records to exclude medical illness that might cause these symptoms.

**Results:**

Of 1443 participants (58.0% response rate) medical records of 990 were examined. 9.4% (N = 93) had unexplained chronic widespread pain and 12.6% (N = 125) had unexplained chronic fatigue. Marital status, childhood psychological abuse, recent threatening experiences and other somatic symptoms were commonly associated with both widespread pain and fatigue. No common effect was found for few years of education and current medical illnesses (more strongly associated with chronic widespread pain) or recent illness in a close relative, neuroticism, depression and anxiety scores (more strongly associated with chronic fatigue). Putative associated factors with a common effect were associated with unexplained chronic widespread pain or unexplained chronic fatigue only when there was concurrent anxiety and/or depression.

**Discussion:**

This study suggests that the associated factors for chronic widespread pain and chronic fatigue need to be studied in conjunction with concurrent depression/anxiety. Clinicians should be aware of the importance of concurrent anxiety or depression.

## Highlights

• Chronic widespread pain and chronic fatigue commonly co-occur, share similar risk factors and are considered by some to be part of the same disorder.
• In this study not all putative associated factors were commonly associated with both disorders.
• The apparent common association appeared to be explained by the presence of common psychiatric disorders, anxiety and depression.

## Introduction

Chronic widespread pain and chronic fatigue are common and may be disabling; they have complex aetiologies [1], [2], [3], [4], [5], [6], [7], [8]. These functional somatic syndromes share common risk factors [9], [10], [11], [12], a finding which has been interpreted as suggesting that chronic widespread pain and chronic fatigue are manifestations of a single disorder [8]. An alternative view is that they are separate syndromes which frequently co-occur and this co-occurrence can be attributed to two dimensions, which have separate genetic and environmental components: an affective component (depression and anxiety) and a sensory component (especially chronic widespread pain) [13], [14]. Comorbid anxiety and depression commonly occur in individuals with chronic fatigue and the risk factors for chronic fatigue differ between those with, and those without, concurrent anxiety or depression [15]. It is plausible that the observation of common associated factors across chronic fatigue and chronic widespread pain is explained by co-morbid anxiety and depression. The aim of this study was to test the hypothesis that the associated factors commonly associated with both chronic widespread pain and chronic fatigue would be explained by the presence of concurrent depression/anxiety.

## Methods

**Fig. 1:**
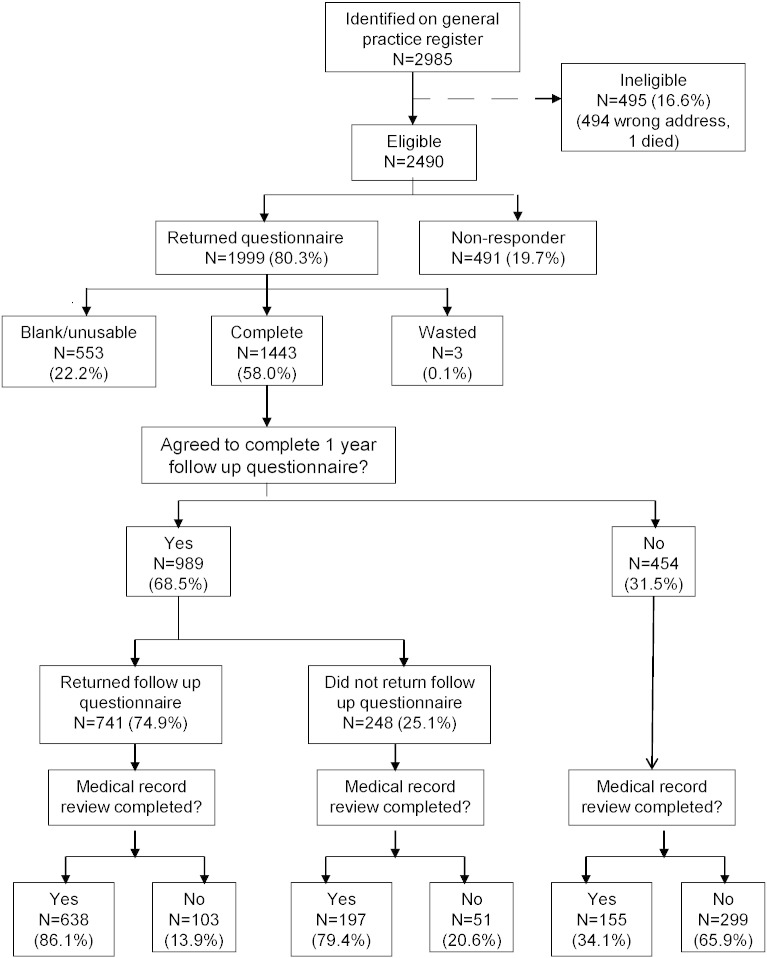
Flow of study participants.

We conducted a cross-sectional population-based study. We mailed 2985 baseline questionnaires to people aged 25–65 years registered at two general practices in North West England, one in an affluent rural area and one in a more deprived inner city area. Potential participants were selected from complete population lists (i.e. GP registers) using simple random sampling assuming that the sampled sub-group was representative of the population from which they were drawn (Fig. 1). Of those 2490 were eligible to participate and were sent a questionnaire that assessed the presence of chronic widespread pain, chronic fatigue and a number of potential associated factors (see below for details).

Written informed consent was sought to examine participant's medical records. The aim of the medical record review was to identify recorded general medical illness that could explain the presence of pain or fatigue and to count the number of consultations over the year prior to questionnaire completion. Non-responders were sent a reminder postcard after two weeks and, if necessary, a further questionnaire after two further weeks.

## Definition of symptom groups

Since our study did not include a medical examination which would enable us to make a specific diagnosis, we refer to the relevant symptoms of pain and fatigue as “symptom groups”.

### Chronic widespread pain

Participants were asked to report the presence of any musculoskeletal pain they had experienced in the past month, whether their pain had persisted for three months or more, and to shade on a four-view blank body manikin the location(s) of their pain. Using these data participants satisfying the criteria for chronic widespread pain included in the American College of Rheumatology 1990 criteria for fibromyalgia (pain above and below the waist, in the right and left hand sides of the body and in the axial skeleton, present for at least three months) [16] were identified.

### Chronic fatigue

The fatigue scale contains 11 items that inquire about symptoms of physical and mental fatigue. Individual items are scored 0 or 1, with a total score ranging from 0 to 11. Participants with fatigue scores of 4 or more on the Fatigue Scale [17] and who had reported symptoms for six months or more were classified as having chronic fatigue.

### Medical record review

For participants who had agreed, medical records were reviewed for 12 months before and after the date of baseline questionnaire by two raters (FC and CCG) to see if there was evidence of a recognised medical condition that could explain chronic fatigue or chronic widespread pain. A conservative approach was used; any medical illness that could cause fatigue or widespread pain led to exclusion from the symptom groups of *unexplained* fatigue or widespread pain so only those participants without such a condition were classified as having chronic fatigue or chronic widespread pain. Nearly half of those who had reported fatigue or widespread pain had consulted their GP with the relevant symptom and, of these, one third had undergone investigations that would be helpful in ruling out underlying organic disease.

## Socio- demographic details

These included age, sex, marital status, current work status (including disability status), number of years of formal education and details of any outstanding compensation claims.

### Co-morbid general medical illness

Respondents were asked if they had any common medical illnesses on a checklist and add any not listed. For analysis, participants were classified as having none, one, two or more general medical illnesses.

### Other bodily symptoms

The Somatic Symptom Inventory (SSI) asks respondents to rate 13 bodily symptoms on a 5-point scale as to “how much it has bothered you over the past 6 months?” The total score ranges from 13 to 65 with high scores indicating greater bother [18].

## Childhood Factors

The *Childhood Physical and Sexual Abuse questionnaire* consists of 8 questions concerning abuse [19]. Respondents were rated as having experienced childhood abuse if, before the age of 16 years, they reported that an older person touched them or they were made to touch someone else in a sexual way, or intercourse was attempted or completed (sexual abuse); that they were hit, kicked or beaten often and/or their life was seriously threatened (physical abuse); they were often insulted, humiliated or made to feel guilty (psychological abuse).

The *Parental Bonding Instrument* includes 7 questions concerning perceived maternal care and 1 item concerning maternal control [20], [21].

## Adult attachment, recent stress and mental state

The *Relationship Scales Questionnaire* measures adult attachment style by asking respondents to identify which of four sets of characteristics most closely matches the way they relate to other people [22]*.* These are: secure (trusting in others), preoccupied (emotionally dependent, low self-esteem), fearful (low trust of others, fearful of intimacy) and dismissing (low trust in others, compulsively self-reliant).

*Social Support* was assessed with a question determining whether the respondent had a close confidant with whom they can discuss all concerns.

*The List of Threatening Experiences* (*LTE-Q*) measures the experience of 12 threatening personal situations or events in the last 6 months [23]. The total score of positive responses represents recent exposure to threatening experiences; we quote the results in 3 groups (0, 1, 2 or more). We also quote separately the scores for questions regarding illness in the participant and close relatives.

The *Revised NEO Personality Inventory* (*NEO-PI-R*) measures the personality trait of Neuroticism [24]. It has a maximum score of 48 with high scores indicating higher levels of neuroticism.

*The Hospital Anxiety and Depression Scale* (*HADS*) is a valid and reliable measure of anxiety and depression in the general population which avoids questions about physical symptoms (e.g. weight loss, pain) that might be caused by general medical illness [25]. A score of 11 or more indicates probable disorder for each dimension but a total HADS score (anxiety + depression) of 17 + has been used also to detect probable depressive disorder [26].

## Health status and healthcare use

The *Short Form 12* (*SF12*) *Questionnaire* assesses health status [27]. It is a validated shortened version of the 36 item version and both versions have been used in chronic fatigue and chronic widespread pain [28], [29], [30], [31] The 12 items yield summary scores for mental (SF12-MCS) and physical (SF12-PCS) components of health status, which are transformed into norm based scoring (27). A low score represents impairment of health status.

### Healthcare use

For participants who had agreed to a review of their medical records we counted all consultations with the general practitioner or practice nurse for 12 months before and after the baseline questionnaire.

The study received ethical approval from the North Manchester Local Research Ethics Committee (REC reference number: 06/Q1406/14). All participants provided written informed consent to participate in the study.

## Statistical analysis

Multi-level modelling was used to take into account that chronic widespread pain and chronic fatigue were measured on each individual, and these symptom groups may not be independent of each other. This technique takes into account that the correlation of symptom groups within individuals will be greater than that between individuals. Each symptom group was thus treated as a within subject factor called ‘type’ with two levels representing the two symptom groups. Other variables measured at the subject level, such as childhood abuse and anxiety and depression (the putative associated factors) were entered in turn into a series of logistic regression analyses using the stata command xilogit, which included age and gender as between subject covariates, and with symptom groups (yes/no) as the dependent variable. Initially, symptom-specific associations were calculated using a population average model. A term for the interaction between ‘type’ and the associated factor was then added to the model, and a Wald test carried out to investigate whether the strength of association of the associated factor was similar across both symptoms, while taking into account within subject correlation of having both symptoms. The Wald test provides p-values to assess the interaction of ‘type’ with the associated factor. Therefore, small p-values (p ≤ 0.05) would indicate that differential effects are likely, while larger p-values (p > 0.05) indicate that a common effect is plausible, in which case the common effect estimate was obtained from the model. Common effect odds ratios are presented only when the Wald test for the interaction between type of disorder and the associated factor was not significant. In this case common effect odds ratios were obtained using the stata command xtlogit with age and gender as covariates, but without the interaction term. Where the interaction was significant ‘no common effect’ has been tabulated, and odds ratios for that associated factor for chronic widespread pain and chronic fatigue separately should be interpreted. These were obtained using the stata command xtlogit with age and gender as additional covariates. Scored variables, SSI, SF-12 mental and physical scores, neuroticism and HADS scores have been split into 3 tertile groups in order to assist in the interpretation of their odds ratios. These analyses were repeated with anxiety, depression and number of general illnesses as covariates in addition to age and gender.

Participants classified as having chronic widespread pain or chronic fatigue were then further divided into those with (HADS score ≥ 17) and without (HADS score < 17) anxiety and/or depression [26]. The associated factors that were observed to be significantly common in both symptom groups were then compared across the three resulting groups (a) symptom plus anxiety and/or depression, b) symptom without anxiety and/or depression and c) no symptom, using the chi-squared test for dichotomous variables and one-way ANOVA for continuous scores, followed by Bonferroni pairwise comparisons between groups. This was then repeated for 4 factors which did not show a common effect across both symptom groups.

## Results

### Participation rates

Of the 2490 questionnaires mailed, 1999 were returned (return rate 80.3%) of which 556 (22.3%) were blank or did not contain usable information (see Fig. 1). The response rate was similar in the two practices (62% inner city and 66.3% rural area). A total of 1443 (58.0%) participants returned a completed questionnaire and participated in the study. Non-responders were significantly more likely to be male (53.1% versus 42.3%), and younger (mean = 43.9 versus 47.0 years) than the remaining eligible participants. The participation rates at the two practises were similar (56.3% and 59.3%).

We examined 990 medical records of the 992 (69%) participants who gave permission for this. Those who refused permission were younger (45.8 v 47.5 years, p = 0.013) and more likely to be female (63.2% v 55.5%, p = 0.008) but did not differ in terms of marital status, years of education, unemployment, prevalence of chronic widespread pain or chronic fatigue by questionnaire or anxiety, depression or somatic symptoms scores. Completed follow up questionnaires were received from 741 (75% of the 989 who agreed), of whom 638 (86.1%) also had their medical notes examined (Fig. 1) but these data are not used in this paper [2].

### Prevalence of each symptom group

After exclusions because of missing data (chronic widespread pain [n = 5] or chronic fatigue [n = 6)]), 159 (11.1%) participants fulfilled criteria for chronic widespread pain and 229 (15.9%) had chronic fatigue. Of the 990 participants with medical record review, the prevalence figures were similar: 11.4% (n = 113, 95% CI 9.5 to 13.4) and 15.5% (n = 153, 95% CI 13.2 to 17.7) respectively, but 20 (17.7%) cases of chronic widespread pain and 28 (18.3%) cases of chronic fatigue could be attributed to a co-existing general medical illness. The prevalence of *unexplained* chronic widespread pain was 9.4% (n = 93, 95% CI 7.6 to 11.2), and chronic fatigue 12.6% (n = 125, 95% CI 10.6 to 14.7) and our analyses concerned these participants who fulfilled criteria for the *unexplained* symptom definitions. Mean SF-12 physical component scores were 42.4 (SD = 10.9) and 43.3 (SD = 11.8) for chronic widespread pain and chronic fatigue, respectively, indicating impaired health status.

### Associated factors and common effects

The majority of the putative associated factors were associated with both chronic fatigue and chronic widespread pain and showed a common effect. The factors associated with a 2 or more fold increased odds across *both* symptom groups included: being separated, widowed or divorced, unemployed and seeking work, reported psychological abuse during childhood, reported physical abuse during childhood, loss of mother at age < 16, experience of a recent serious illness or injury, two or more recent threatening experiences, and a high somatic symptom score (Table 1). Frequent consultations in primary care and a low SF-12 physical component score (indicating impairment) were common to both symptom groups.

A number of factors showed no common effect. Fewer than 12 years of formal education and 2 or more current general medical illnesses were both more strongly associated with chronic widespread pain than with chronic fatigue. Recent serious illness or injury to a close relative was strongly associated with the presence of chronic fatigue but not chronic widespread pain. There was also no common effect of neuroticism, depression, anxiety and SF-12 mental component scores with the stronger relationship observed for those participants with chronic fatigue.

After adjusting for anxiety, depression and number of general medical illnesses, in addition to age and gender, these results remained similar.

### Association with anxiety and depression

The proportion of participants with concurrent anxiety and depression (HADS total score of 17 or more) was 41.6% of participants with chronic fatigue (52/125) and 24.7% of those with chronic widespread pain (23/93), p = 0.010.

### *Concurrent depression*/*anxiety*

#### *Associated factors with common effect*

**Fig. 2:**
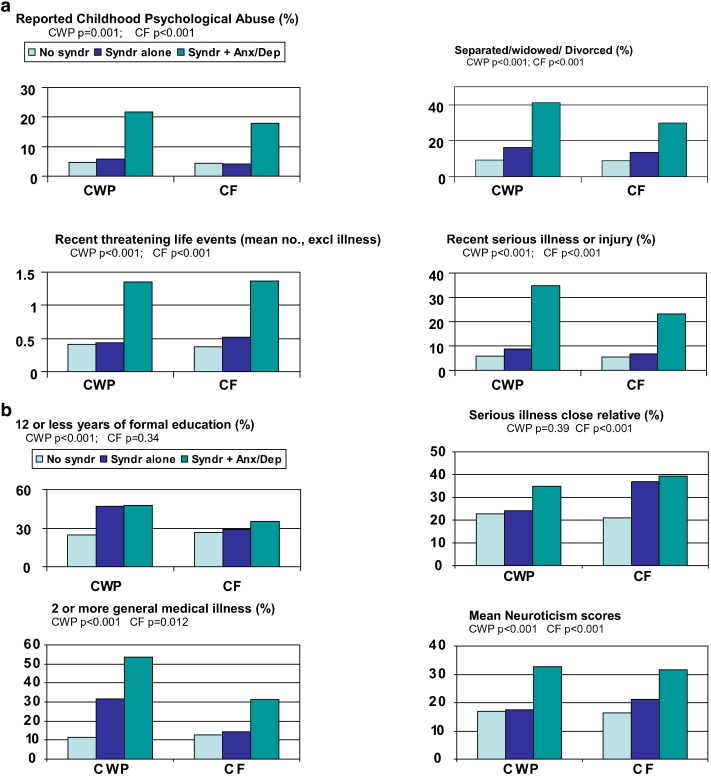
a comparison of symptom groups with and without anxiety/depression: risk factors with a common effect across the symptoms. Fig. 2b comparison of symptom groups with and without anxiety/depression: risk factors without a common effect across the symptoms.

The putative associated factors which showed a common effect (childhood psychological abuse, separated/widowed/divorced, recent serious illness/injury and 2 or more threatening life events) were more common in participants with chronic fatigue or widespread pain who reported concurrent anxiety and/or depression compared to participants with these symptoms alone (Fig. 2a). Approximately 5% of participants with chronic widespread pain or chronic fatigue *without* concurrent anxiety and/ordepression reported psychological abuse, which was similar to participants free of chronic widespread pain or chronic fatigue and significantly fewer than participants with these symptoms plus concurrent anxiety and/or depression (approximately 20%). A similar pattern was found with the other putative associated factors that had a common effect (Fig. 2a).

#### *Associated factors with no common effect*

The pattern of association was different for putative associated factors with no common effect (Fig. 2b). Nearly half of participants with chronic widespread pain had received 12 or fewer years of formal education, whether or not there was concurrent anxiety and/or depression; this compared to a quarter of participants without chronic widespread pain. In chronic fatigue there was no significant difference in duration of education between the 3 groups (Fig. 2b).

Over half of participants (53.8%) with chronic widespread pain and concurrent anxiety and/or depression had 2 or more recognised general medical illnesses; this compared with 32% of those with chronic widespread pain without anxiety and/or depression, and 11% of those without chronic widespread pain. Of participants with chronic fatigue and anxiety and/or depression 31.2% had 2 or more general medical illnesses compared to 14.3% of those with chronic fatigue alone and 13% of participants without chronic fatigue.

Recent serious illness or injury in a close relative was reported more frequently by participants with chronic fatigue, whether or not they had concurrent anxiety and/or depression (approximately 38%) compared to those without the symptom groups (21.0%). There was no significant difference in chronic widespread pain (Fig. 2b).

Mean neuroticism scores in participants with chronic widespread pain without anxiety or depression were similar to those free of chronic widespread pain (17.0 [SD = 9.1] and 17.4 [7.5]); this score was lower than that for participants with chronic widespread pain with concurrent anxiety and/or depression (32.6 [6.7]). Participants with chronic fatigue alone, on the other hand, had a mean neuroticism score significantly different from those without this symptom (21.2 [SD = 8.1] v 16.3 [8.7]: p < 0.001 Bonferroni) (Fig. 2b).

## Discussion

### Summary of main findings in the context of current knowledge

This is the first study to show that the putative associated factors for chronic fatigue and chronic widespread pain were not associated with each symptom in an identical fashion. The factors which appear to be common to each of these were only associated with them when there was also concurrent anxiety and depression. For example, although fatigue and chronic widespread pain each showed an association with reported childhood psychological abuse, this could be attributed to the presence of anxiety or depression rather than a true correlate of the fatigue or widespread pain.

Similar findings were reported in a birth cohort study where adjustment for psychopathology led to childhood physical abuse becoming non-significant as a risk marker of CFS-like illness [1]. Rather similar effects were found in a study of widespread pain: adjustment for PTSD led to the prior experience of witnessing a traumatic event becoming non-significant [32]. We found also that threatening life events were associated with chronic fatigue and widespread pain only in the presence of concurrent anxiety or depression; the association with chronic fatigue has been reported previously in two prospective cohort studies [15], [33], [34]. This pattern of associations also held for previously married status and reported childhood psychological abuse in both chronic fatigue and chronic widespread pain. Our finding that neuroticism scores were raised in participants with chronic fatigue, whether or not there was accompanying anxiety and/or depression is similar to that concerning chronic fatigue in one birth cohort study [15].

Our results extend those of our previous study of common associated factors across these symptom groups [10] because we widened the range of possible associated features and found new features that did not have a common effect - duration of education, current general medical illnesses, having an ill relative and neuroticism. Although they were shown to have a common effect, depression and anxiety were much more closely associated with chronic fatigue than the other symptoms groups in our previous study [10].

The association between chronic widespread pain and few years of education and general medical illness appears to be independent of psychiatric disorder. This has been reported previously but ours is the first demonstration of the contrast between chronic widespread pain and chronic fatigue in this respect [35], [36]. Whether the relationship with few years of education is a specific or general effect is not known [37].

### Strengths and limitations

Our study has a number of strengths as it used well-recognised case definitions of chronic fatigue and chronic widespread pain in a population-based sample rather than self-described chronic fatigue or attenders at primary care [15], [38]. We excluded cases where the fatigue or pain could be explained by recognised organic disease, which has been done only in some previous population-based studies. On the other hand, we did not use an interviewer-based detailed definition of chronic fatigue preventing us from extrapolating our findings to this smaller group of the more severe chronic fatigue syndrome. This is important as childhood physical abuse was an associated factor for chronic fatigue syndrome/ME in the cohort study which did not find this association in CFS-like illness once psychopathology was adjusted for [1]. This suggests subtle differences according to the symptom group studied and the way associated factors and psychopathology are defined and measured [3], [39], [40]. It is worth noting that chronic fatigue is much more common and relevant to primary care, than chronic fatigue syndrome [41]. We also relied on a self-administered questionnaire to assess childhood abuse and this may not be the most reliable method.

Our study was limited as our main analysis was cross-sectional, preventing true assessment of risk factors. Larger prospective studies, however, have found also that neuroticism and depression are predictors of subsequent chronic fatigue [15], [42]. Others found that few years of education and one or more longstanding physical disease predicted later onset of chronic pain [35].

### Interpretation of our results

Although our method was quite different, our findings support the suggestion from twin studies that concurrence of functional somatic syndromes can be explained, in part, by two latent traits — one primarily psychiatric and one sensory or pain component [11], [13]. We found no association between reported childhood psychological abuse and chronic widespread pain or fatigue in the absence of anxiety or depression, suggesting that this is not a true associated factor for these symptom groups but only applicable when there is concurrent anxiety and/or depression [33], [44], [45]. This may explain why results concerning sexual abuse as a common associated factor for chronic fatigue syndrome are inconsistent [33], [43], [46].

Since our study was cross-sectional we cannot comment on the temporal relationship between chronic fatigue or widespread pain and anxiety and/or depression but others have found that depression precedes fatigue and vice versa [33], [34], [42]. It is most likely that there are different pathophysiological pathways to chronic fatigue syndrome [33], [38], [47]. Our data suggest that some of the putative associated factors for chronic fatigue and chronic widespread pain are, in fact, associated factors for the concurrent anxiety or depression frequently observed with these symptoms. It is possible however that anxiety or depression may represent one pathway to chronic fatigue, in particular.

The implications of our study are twofold. From the research perspective, our difficulty in understanding the aetiology of the functional somatic syndromes will remain while the cause of each symptom group or syndrome is sought as a single entity. Instead, our data suggest that the search for causes should look at common aetiological factors across different functional somatic syndromes, notably those associated with psychiatric disorders, simultaneously with the unique associated factors for each syndrome [48]. Another, similar approach is to compare the aetiological pathways of multiple somatic symptoms and multiple syndromes with those of discreet syndromes [48].

From the clinical perspective, it is helpful for clinicians and patients to know that the presence of a chronic fatigue or chronic widespread pain does not necessarily imply a history of abuse or psychiatric disorder. Such implications may get in the way of satisfactory consultations and care. On the other hand it should be routine that clinicians explore these issues with all patients who have a functional somatic syndrome, including case-finding for anxiety and depression, and discuss appropriate management options if relevant. Current evidence suggests that separate treatments for somatic symptoms and psychiatric symptoms are helpful. The former often involves specific cognitive behaviour therapy aimed at beliefs related to somatic symptoms and/or some form of exercise [49], [50], [51], [52]; the latter often involves a psychological treatment for anxiety or depression and/or antidepressant therapy as described in NICE guideline [53].

## Author contributions

Authors made substantial contributions in the areas outlined below. In addition all authors discussed the results, drafted and/or revised the article critically and have given final approval of this version to be submitted for publication. The corresponding author takes responsibility for the integrity of the data and the accuracy of the data analysis.

**Chew-Graham, Creed, Macfarlane, McBeth**: Study development, design, data collection, data analysis, manuscript preparation and revision.

**Davies, Jackson, Littlewood**: Data collection and analysis, manuscript preparation and revision.

**Tomenson B**: Data analysis, manuscript preparation and revision.

## Conflicts of Interest

None of the authors have conflicts of interest to report.

## Figures and Tables

**Table 1:** Individual and common effects between each putative risk factor and MUS (n = 990) ^1^, adjusted for age and sex. Odds ratios and 95% confidence intervals.

	CWP (n = 93) vs no CWP (n = 897)		CF (n = 125) vs no CF (n = 865)		Comparison	Common effect	
OR	95% CI	OR	95% CI	p	OR	95% CI
*Demographic details (N, %)*							
Age:							
25–34 (166.17%)	1.0	Referent	1.0	Referent	1.0	Referent
35–44 (259,26%)	1.4	0.7–2.9	1.1	0.6–2.0	0.20	1.2	0.7–2.0
45–54 (238.24%)	0.9	0.4–2.0	0.9	0.5–1.6		0.9	0.5–1.5
≥ 55 (327.33%)	1.8	0.9–3.6	0.9	0.5–1.6	1.2	0.8–1.9
Female sex^2^ (549.56%)	1.1	0.7–1.7	1.2	0.8–1.8	0.69	1.1	0.8–1.6
Single (148.15%)	0.8	0.4–1.6	1.1	0.6–1.9	0.22	1.0	0.6–1.5
Married/cohabiting (732.74%)	1.0	Referent	1.0	Referent	-	1.0	Referent
Separated/widowed/divorced (102.10%)	2.7	1.5–4.8	2.7	1.3–5.7	0.80	2.7	1.8–4.0
Less than 12 years of education (261.26%)	2.6	1.5–4.4	1.4	0.9–2.2	0.01	No common effect	
No confidant (64.6%)	1.4	0.6–3.1	3.1	1.6–6.0	0.09	2.2	1.3–3.6
Working (778.79%)	1.0	Referent	1.0	Referent	-	1.0	Referent
Unemployed, and seeking work (18.2%)	2.2	0.6–7.7	2.7	0.6–13.1	0.69	2.4	1.0–5.9
Not working due to ill health (26.3%)	1.8	0.0–230	2.0	1.2–3.5	0.56	1.9	1.2–2.9
Student, retired, etc. (152.15%)	0.8	0.4–1.5	0.8	0.4–1.5	0.51	0.8	0.5–1.3
Off work due to ill health in the past month (111.11%)	1.9	1.1–3.4	2.3	1.4–3.8	0.53	2.1	1.4–3.1
Compensation claim (10.1%)	6.8	1.8–25.2	1.8	0.4–8.8	0.14	3.5	1.2–10.1

*Health status and healthcare use*							
Somatic Symptom Index score:							
< 26 (787.80%)	1.0	Referent	1.0	Referent		1.0	Referent
26–30 (115.12%)	4.0	2.2–7.2	5.2	2.9–9.0	0.72	4.4	3.0–6.5
> 30 (88.9%)	8.9	4.9–16.4	11.0	5.7–21.0		9.3	6.3–13.7
2 or more current medical illnesses (88.9%)	4.5	2.3–8.7	2.0	1.1–3.8	0.017	No common effect	
SF-12 mental score:							
≥ 50 (558.56%)	1.0	Referent	1.0	Referent			
40–50 (217.22%)	1.2	0.7–2.2	3.3	1.7–6.3	< 0.001	No common effect	
< 40 (215 (22%)	2.9	1.7–4.8	16.0	5.8–44.4			
SF-12 physical score:							
≥ 50,657.66%)	1.0	Referent	1.0	Referent		1.0	Referent
40–50 (186.19%)	3.4	1.9–5.9	2.1	1.3–3.5	0.72	2.5	1.7–3.7
< 40 (147.15%)	5.3	3.0–9.4	4.7	2.9–7.8		4.7	3.3–6.8
No of consultations in primary care in the previous year:							
0 (289.29%)	1.0	Referent	1.0	Referent		1.0	Referent
1 (203.21%)	1.4	0.7–2.7	2.1	1.1–3.9	0.60	1.7	1.1–2.8
2 or 3 (249.25%)	1.3	0.7–2.5	2.2	1.2–4.0		1.8	1.2–2.7
≥ 4 (187.19%)	2.6	1.4–4.8	3.1	1.6–5.8		2.8	1.8–4.4

*Childhood factors*							
Maternal care score:							
≥ 18 Good (522.53%)	1.0	Referent	1.0	Referent		1.0	Referent
15–18 Poor (222.22%)	1.3	0.8–2.2	1.2	0.7–1.9	0.19	1.2	0.8–1.8
0–14 Very poor (246.25%)	1.0	0.6–1.7	1.6	1.1–2.6		1.3	0.9–1.9
Maternal over-control	0.95	0.6–1.5	1.4	0.9–2.0	0.20	1.2	0.8–1.6
Any childhood abuse (65.7%)	1.9	0.9–4.1	2.0	0.98–4.0	0.84	1.9	1.2–3.2
Sexual abuse (113.11%)	1.6	0.8–2.9	1.9	1.2–3.2	0.55	1.7	1.1–2.7
Psychological abuse (51.5%)	2.3	1.05–5.2	2.3	1.1–4.8	0.92	2.2	1.3–3.9
Physical abuse (35.4%)	2.1	0.8–5.7	2.2	0.9–5.4	0.96	2.1	1.1–4.0
Loss of mother < 16 yrs. (22.2%)	2.1	0.7–6.5	2.1	0.7–6.3	0.94	2.1	0.9–4.7
Loss of father < 16 yrs. (52.5%)	2.1	0.9–4.7	0.9	0.4–2.2	0.10	1.4	0.7–2.5

*Adult attachment, recent stress and current mental state*							
Fearful attachment (143.14%)	1.5	0.9–2.7	2.5	1.5–4.0	0.12	2.0	1.4–2.9
Preoccupied attachment (71.7%)	1.3	0.6–2.8	2.4	1.3–4.3	0.15	1.9	1.1–3.1
Dismissing attachment (216.22%)	1.5	0.92–2.5	0.9	0.5–1.4	0.046	No common effect	
Recent serious illness or injury to the participant (65.7%)	3.0	1.5–5.8	2.8	1.5–5.3	0.82	2.8	1.8–4.5
Recent serious illness or injury to a close relative (228.23%)	1.2	0.8–2.0	2.3	1.5–3.5	0.034	No common effect	
Death of close relative (51.5%)	1.5	0.7–3.6	2.8	1.5–5.5	0.23	2.2	1.3–3.8
Death of close friend (158.16%)	1.6	0.9–2.6	1.6	0.96–2.6	0.88	1.5	1.1–2.3
Threatening experiences:							
None (478.48%)	1.0	Referent	1.0	Referent		1.0	Referent
One (278.28%)	1.4	0.8–2.3	1.9	1.2–3.1	0.16	1.6	1.1–2.4
Two or more (232.23%)	2.2	1.3–3.6	4.0	2.5–6.3		3.0	2.1–4.3
Neuroticism score:							
0–11 (270.27%)	1.0	Referent	1.0	Referent			
12–17 (261.26%)	1.8	0.9–3.7	1.9	0.8–4.8			
18–24 (232.23%)	2.3	1.1–4.6	5.8	2.2–15.3	< 0.001	No common effect	
> 24 (227.23%)	3.4	1.7–6.8	16.6	3.7–73.7			
HADS anxiety score:							
0–7 (664.67%) 8–10 (185.19%) ≥ 11 (141.14%)	1.0 2.0 3.9	Referent 1.2–3.5 2.2–6.9	1.0 4.1 9.3	Referent 2.5–6.9 5.3–16.4	0.011	No common effect	
HADS depression score:							
0–7 (900.91%)	1.0	Referent	1.0	Referent			
8–10 (56.6%)	2.9	1.4–6.2	6.0	3.0–11.9	0.031	No common effect	
≥ 11 (34.3%)	3.7	1.5–8.8	11.5	4.7–28.1			

1 Excludes subjects with missing data.

2 adjusted for age only.

MUS Medically Unexplained Symptoms.

CWP = chronic widespread pain, CF = chronic fatigue.

OR = odds ratio, 95% CI = 95% confidence interval for odds ratio.

P = p-value for Wald test for common effects. Common effect odds ratio and 95% CI is only presented if Wald p is not significant at the 5% level.
